# Laparoscopic vs. Open-Groin Hernia Repair in Romania—A Populational Study

**DOI:** 10.3390/jcm14082834

**Published:** 2025-04-19

**Authors:** Nicolae Dragos Garofil, Mihai Zurzu, Mircea Nicolae Bratucu, Vlad Paic, Anca Tigora, Cristian Vladescu, Silviu Badoiu, Victor Dan Eugen Strambu, Petru Adrian Radu, Sandu Ramboiu

**Affiliations:** 1 Faculty of Medicine, “Carol Davila” University of Medicine and Pharmacy, 050474 Bucharest, Romania; dragos.garofil@umfcd.ro (N.D.G.); mircea.bratucu@umfcd.ro (M.N.B.); vlad.paic@drd.umfcd.ro (V.P.); anca.tigora@drd.umfcd.ro (A.T.); victor.strambu@umfcd.ro (V.D.E.S.); petru.radu@umfcd.ro (P.A.R.); 2 National Institute of Health Services Management, 030167 Bucharest, Romania; cvladescu@inmss.ro; 3 Sixth Department of Surgery, Craiova Emergency Clinical 7 Hospital, University of Medicine and Pharmacy of Craiova, 200642 Craiova, Romania

**Keywords:** inguinal, groin, hernia, laparoscopic repair, open repair, minimally invasive surgery, DRG

## Abstract

**Background/Objectives:** Groin hernia repair is a common surgical procedure worldwide, with increasing adoption of minimally invasive techniques. However, the adoption of laparoscopic repair varies significantly across healthcare systems. This study aims to analyze trends in laparoscopic versus open-groin hernia repair in Romania over a five-year period (2019–2023), assessing differences in hospital types, reimbursement policies, and patient outcomes. **Methods:** This nationwide retrospective study examined 76,553 groin hernia repairs from the National Diagnosis-Related Group (DRG) database, including 231 public and 41 private hospitals. Patients were categorized as laparoscopic (13,282 cases) or open repair (63,271 cases). Statistical analysis included logistic regression and non-parametric tests to assess factors influencing surgical approach selection, hospitalization duration, and case complexity. **Results**: Laparoscopic repair accounted for 17.3% of all groin hernia procedures, with higher adoption in private hospitals (54.7%) than in public hospitals (14.6%). Laparoscopic procedures increased from 14.1% in 2019 to 20% in 2023. Hospitalization was shorter in private hospitals (1.78 vs. 4.80 days in public hospitals). Reimbursement rates showed minimal differentiation between laparoscopic and open repair, suggesting no financial incentive for minimally invasive surgery in public hospitals. **Conclusions**: Despite a steady increase in laparoscopic hernia repair, its adoption in Romania remains limited compared to Western Europe. Private hospitals lead in minimally invasive surgery, while public hospitals predominantly rely on open repair due to reimbursement policies and resource constraints. Adjusting DRG-based reimbursement, expanding training, and implementing a national hernia registry could improve outcomes and access to minimally invasive surgery.

## 1. Introduction

Groin hernia repair is one of the most frequently performed elective surgical procedures worldwide [1]. This condition affects a significant percentage of the population, and surgical intervention is often necessary due to discomfort, the risk of complications, and its negative impact on patients’ quality of life. Although most procedures are elective, between 2% and 7% of cases require emergency surgery due to complications such as incarceration or strangulation [2,3].

The surgical approach to inguinal hernia repair in adults has evolved significantly, transitioning from traditional herniorrhaphy to tension-free, mesh-based techniques and, more recently, to minimally invasive procedures such as the transabdominal preperitoneal (TAPP) and totally extraperitoneal (TEP) laparoscopic approaches [4,5]. Similarly, in the pediatric population, there has been a notable shift from open to laparoscopic techniques, with percutaneous internal ring suturing (PIRS) becoming particularly popular among pediatric surgeons due to its minimal invasiveness and favorable outcomes [6]. Minimally invasive techniques offer several advantages, including reduced postoperative pain, faster recovery, shorter hospital stays, and lower rates of wound-related complications [7]. However, the adoption of laparoscopic surgery remains inconsistent and is influenced by factors such as equipment availability, surgeon experience, associated costs, and healthcare funding policies [8].

In Romania, the healthcare system includes both public and private hospitals, each with different approaches to managing patients with inguinal hernias. Private hospitals, supported by flexible financing structures and direct patient payments, are better positioned to adopt new technologies rapidly, whereas public hospitals are often constrained by financial and logistical limitations. However, public hospitals remain the primary providers of healthcare services for the majority of the population, especially for patients who cannot afford private-sector interventions [9].

International studies have shown that the adoption of laparoscopic techniques is influenced by healthcare policies and medical infrastructure. For example, data from Spain and Italy indicate a steady increase in the use of minimally invasive approaches, although their adoption remains below international recommendations [8,10]. On the other hand, Denmark has largely adopted laparoscopic hernia repair as the preferred approach, and data from their national hernia registry show that this widespread adoption has led to significant improvements in recurrence rates and postoperative outcomes [11].

The COVID-19 pandemic significantly impacted surgical activity, leading to a sharp decline in the number of elective procedures in public hospitals, while the private sector managed to maintain relatively stable surgical volumes [12,13]. This situation has exacerbated disparities between the two healthcare sectors and highlighted the need for reforms to improve access to minimally invasive procedures.

Our previous research on the nationwide prevalence of groin hernia repair in Romania during the pandemic provided a foundational understanding of surgical trends and healthcare resource allocation [9]. Building upon these findings, this study aims to provide a detailed analysis of inguinal hernia treatment in Romania over a five-year period (2019–2023), comparing surgical activity in public and private hospitals. A key objective of this study is to evaluate the progress in adopting minimally invasive techniques and to identify trends in laparoscopic hernia repair. This study offers a detailed picture of how groin hernia cases are managed in Romania, highlighting institutional differences and opportunities for improvement.

## 2. Material and Methods

### 2.1. Patients

This nationwide retrospective study analyzed anonymized data from 76,553 adult patients (age ≥18 years) who underwent groin hernia repair surgeries in Romania between January 2019 and December 2023. Data were collected from the DRG database provided by the National Institute of Health Services Management (INMSS), encompassing all 272 hospitals (231 public and 41 private hospitals) performing groin hernia repair surgeries during this period.

**Inclusion Criteria:**

- Adult patients (≥18 years);
- Elective or emergency surgery for groin hernias (inguinal or femoral);
- Procedures performed in public or private hospitals and documented within the DRG database.

**Exclusion Criteria:**

- Patients younger than 18 years;
- Surgical interventions not clearly classified using the specified ICD-10 procedure codes.

The procedures analyzed were identified using the following ICD-10 surgical procedure codes: J12701 (Laparoscopic repair of unilateral femoral hernia), J12702 (Laparoscopic repair of bilateral femoral hernia), J12703 (Open surgical repair of unilateral femoral hernia), J12704 (Open surgical repair of bilateral femoral hernia), J12601: Laparoscopic repair of unilateral inguinal hernia, J12602 (Laparoscopic repair of bilateral inguinal hernia), J12603 (Open surgical repair of unilateral inguinal hernia), and J12604 (Open surgical repair of bilateral inguinal hernia).

Patients were classified based on surgical technique into two groups:

- Open repair group (63,271 cases);
- Laparoscopic repair group (13,282 cases).

Demographic and clinical parameters were collected, including hospital type (public or private), patient age, sex, type of hernia (inguinal vs. femoral), recurrence status, presence of obstruction or gangrene, hernia laterality (unilateral vs. bilateral), obesity, and relevant comorbidities.

### 2.2. Ethical Aspects

This study was conducted according to the guidelines of the Declaration of Helsinki (1964) and its subsequent amendments, ensuring patient data confidentiality and ethical compliance. Ethical approval was obtained from the Ethics Committee of the Carol Davila Clinical Nephrology Hospital (Approval number: 90/21.03.2025). Due to the retrospective and anonymized nature of the data used, informed consent was not required.

### 2.3. Study Outcomes

The primary outcome of this study was to identify factors influencing the selection of laparoscopic versus open-groin hernia repair in public and private healthcare settings in Romania.

Secondary outcomes included the proportion and annual trends of laparoscopic versus open procedures, length of hospital stay (preoperative and postoperative), patient demographics and clinical factors influencing surgical approach, geographic variations in laparoscopic adoption across Romanian counties, and comparative rates of hernia recurrence and postoperative mortality between laparoscopic and open repair.

### 2.4. Study Design

This retrospective, observational, nationwide population-based study utilized comprehensive administrative data collected continuously over five years (2019–2023). Data were extracted from the Romanian National DRG database, encompassing both public and private healthcare institutions.

The study utilized patient-level administrative and clinical data, comparing laparoscopic and open surgical techniques with respect to healthcare delivery and patient outcomes.

### 2.5. Statistical Analysis

Descriptive statistics summarized patient characteristics and procedural data. Categorical variables were analyzed using the Chi-square test.

Logistic regression analysis was performed to identify significant predictors influencing the selection of laparoscopic repair, calculating odds ratios (ORs) and corresponding 95% confidence intervals (CIs) for each independent variable. Logistic regression models were stratified separately for public and private hospitals to investigate possible differences in patient selection patterns.

Given the non-normal distribution of continuous data (as confirmed by the Shapiro–Wilk test, *p* < 0.05), non-parametric analyses were conducted. Mann–Whitney U tests compared differences in hospitalization duration, preoperative and postoperative stays between surgical techniques (laparoscopic vs. open) and hospital types (public vs. private). The interaction between surgical technique and hospital type was evaluated using the Kruskal–Wallis test.

Statistical significance was established at *p* < 0.05. All statistical analyses were performed using Python (version 1.15.2, SciPy package).

## 3. Results

Of the 230 public hospitals where groin hernia repair is performed, 152 also carry out laparoscopic procedures, while among the 41 private hospitals, 33 utilize this minimally invasive technique.

Table 1 presents the total distribution of groin hernia repair procedures over the five-year analysis period, differentiated by surgical approach and hospital type. It is evident that procedures performed in public hospitals account for the majority of cases (93.1% of the total), while private hospitals contribute only 6.9% of the total volume of procedures. These figures do not include procedures fully funded outside the National Health Insurance House (NHIH), such as those paid for out-of-pocket in private hospitals, as privately funded procedures are not reported. However, the number of such cases is presumed to be relatively low.

The open approach remains the predominant technique, used in 82.7% of cases, while laparoscopy accounts for 17.3% of procedures. The gap is particularly striking in public hospitals, where only 14.6% of cases were found to be laparoscopic, compared to 54.7% in private hospitals, suggesting a faster adoption of minimally invasive techniques in the private sector, while public hospitals continue to rely mainly on open surgery.

**Figure 1:**
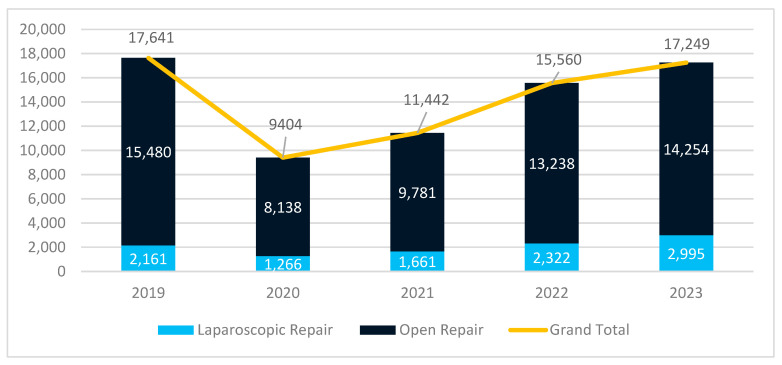
Evolution of surgical procedures in public hospitals.

A progressive increase in the use of laparoscopy was observed between 2019 and 2023 in both public and private hospitals. The COVID-19 pandemic had a significant impact on surgical activity, particularly in public hospitals, where the total number of hernia repairs dropped by 46.7% in 2020 due to hospital restrictions and resource reallocation. A steady recovery followed, with an annual increase of 27.8% between 2020 and 2023, bringing surgical volumes in 2023 close to pre-pandemic levels (Figure 1).

**Figure 2:**
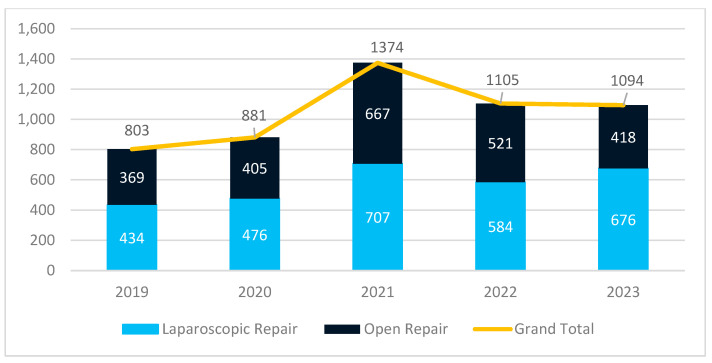
Evolution of surgical procedures in private hospitals.

The private sector followed a different trajectory. Unlike public hospitals, where surgeries sharply declined in 2020, the number of procedures in private hospitals remained stable and even showed a slight increase that year. The largest surge occurred in 2021, with a 71.1% rise compared to 2019, likely driven by patients opting for private care due to limited access in public hospitals. However, after this peak, surgical volumes stabilized in 2022 and 2023, with a 36.2% increase compared to pre-pandemic levels (Figure 2).

A notable trend was the continued increase in laparoscopic repairs, seen in both healthcare sectors. In public hospitals, laparoscopic procedures grew by 38.6% compared to 2019, confirming a gradual shift toward minimally invasive techniques. The increase was even greater in private hospitals, where the number of laparoscopic repairs in 2023 was 55.8% higher than that in 2019, further reinforcing the faster adoption of laparoscopy in private settings.

Groin hernias account for 95.9% of all cases, with 73,386 procedures, while femoral hernias represent only 4.1%, totaling 3167 cases. The minimally invasive approach is used significantly more frequently for inguinal hernias (17.7%, or 13,009 cases) than for femoral hernias (8.6%, or 273 cases).

As expected, groin hernias are much more common in men, who account for 89% of all patients operated on, while women represent only 11% of cases. However, the gender distribution varies depending on the type of hernia. While men are the majority in inguinal hernias, femoral hernias are much more common in women, with 78% of operated patients being female.

Regarding the laparoscopic approach, it was used less frequently in women compared to men. Only 13.3% of procedures in women were performed laparoscopically, compared to 17% in men. This discrepancy is also evident in the private sector, where 46% of female patients underwent laparoscopic surgery, compared to 55% of male patients. These differences may be influenced by anatomical factors, case complexity, or surgical preferences, as laparoscopy is more commonly used for inguinal hernias, which are predominantly seen in men.

**Figure 3:**
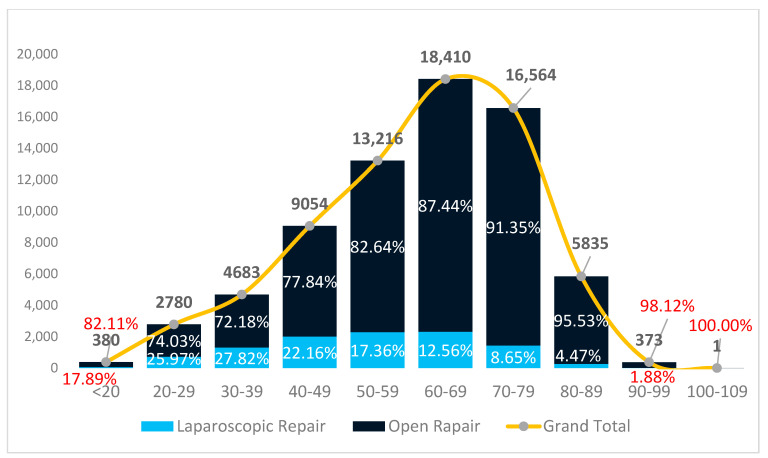
Distribution of patients undergoing groin hernia repair by age group in public hospitals and the percentage of laparoscopic procedures.

As shown in Figure 2 and Figure 3, the age distribution is similar in public and private hospitals, with the highest number of procedures performed in patients aged 60–69, followed by those aged 70–79 and 50–59 years.

However, laparoscopy usage declines with age in public hospitals, where 27.8% of patients aged 30–39 underwent laparoscopic surgery; the rate drops steadily to 12.6% in those over 60 and just 4.5% in patients over 80. This trend indicates a more cautious application of laparoscopy in older patients, potentially due to increased comorbidity or procedural complexity.

In contrast, private hospitals maintain a high and stable rate of laparoscopic repairs across all age groups. Over 60% of patients under 50 underwent laparoscopic surgery, and even in patients over 70, the rate remained above 45%, this being significantly higher than that in public hospitals. Even among octogenarians, laparoscopy was performed in 34.5% of cases, compared to just 4.5% in the public sector.

When analyzing laparoscopic procedures for highly complex hernias, we observe that for incarcerated and recurrent hernias, there are no notable differences between public and private hospitals. The proportion of laparoscopic procedures for incarcerated hernias is relatively similar, with a slightly higher percentage in public hospitals (22.08%) compared to private hospitals (18.8%). Similarly, for recurrent hernias, laparoscopic reinterventions are reported at comparable rates, with 4.53% in the public system and 3.48% in the private sector.

In contrast, a significant difference emerges when looking at bilateral hernia repairs performed laparoscopically. In private hospitals, 17.27% of patients underwent a bilateral approach, meaning that almost one in five patients had both hernias repaired in a single minimally invasive procedure. In the public system, this approach is far less common, with a rate of only 4.81%. This disparity indicates a greater inclination in the private sector toward comprehensive laparoscopic interventions (Table 2).

### 3.1. Factors Influencing the Selection of Laparoscopic Repair

To assess whether there was a patient selection bias influencing the choice of laparoscopic versus open repair, we performed a logistic regression analysis, examining how patient characteristics, hernia type, and hospital setting impacted the likelihood of undergoing a minimally invasive approach.

The logistic regression analysis revealed several key factors that influenced whether patients underwent laparoscopic or open surgery for groin hernia repair. Age emerged as a significant predictor: older patients were significantly less likely to undergo laparoscopic repair, with the effect being stronger in public hospitals (OR = 0.97, *p* < 0.001) than in private hospitals (OR = 0.98, *p* < 0.001), suggesting a more conservative approach to elderly patients in the public sector.

Bilateral hernias were the strongest predictor for laparoscopy, being more than twice as likely to be treated laparoscopically in both settings (OR = 2.25 in public, OR = 2.18 in private, *p* < 0.001), aligning with the advantages of single-access bilateral repairs in laparoscopy.

Femoral hernias were less frequently treated laparoscopically, especially in public hospitals (OR = 0.68, *p* < 0.001), while the association was not significant in private hospitals (*p* = 0.074).

Recurrent hernias showed a notable contrast—public hospitals favored laparoscopy for recurrences (OR = 1.21, *p* < 0.001), whereas private hospitals preferred open repair (OR = 0.53, *p* < 0.001), possibly reflecting different surgical strategies or reimbursement policies.

Obstruction and gangrene significantly reduced the likelihood of laparoscopy, with public hospitals showing an even stronger preference for open repair in these cases (OR = 0.64 for obstruction, OR = 0.84 for gangrene). These findings likely reflect the greater urgency and technical challenges of laparoscopic repair in complicated cases.

Obesity had no significant impact in public hospitals (*p* = 0.206), but in private hospitals, obese patients were significantly less likely to undergo laparoscopy (OR = 0.75, *p* = 0.037), potentially due to longer operative times and increased technical difficulty.

### 3.2. Territorial Distribution of Cases

**Figure 4:**
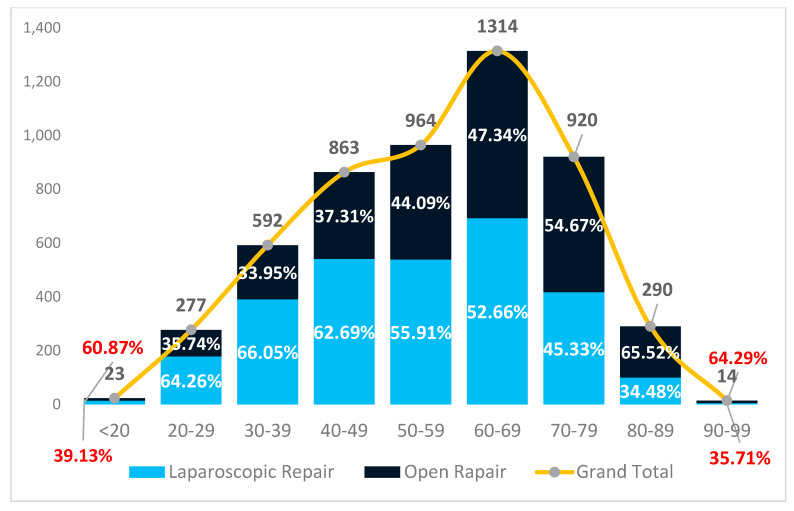
Distribution of patients undergoing groin hernia repair by age group in private hospitals and the percentage of laparoscopic procedures.

Laparoscopic procedures have been introduced in almost all counties, yet their implementation remains uneven. As shown in Figure 4, the highest numbers were recorded in major university centers, with Bucharest (965 cases), Cluj (244 cases), and Timiș (149 cases) leading. However, in some counties, laparoscopy is nearly absent. For instance, in Giurgiu, where only 22 hernia patients were operated on, just 1 patient underwent a laparoscopic procedure, while Vaslui (2 cases), Ialomița (3 cases), and Călărași (4 cases) reported extremely low numbers.

The low adoption of laparoscopy in many areas suggests either a preference for open surgery or limitations in infrastructure, surgeon training, or laparoscopic equipment availability. Nevertheless, its presence even in low-volume counties indicates a gradual nationwide acceptance, though at a highly variable pace.

**Figure 5:**
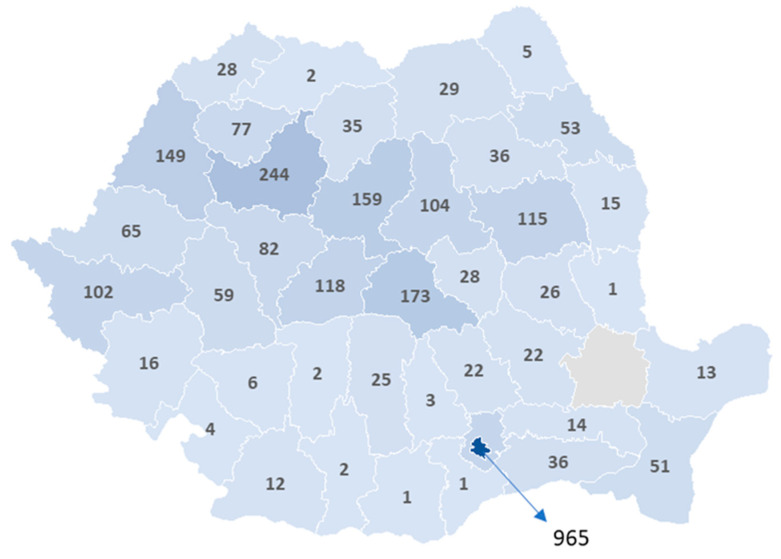
Territorial distribution of laparoscopic groin hernia repair case in public hospitals in 2023.

The private healthcare system exhibits an even more uneven distribution, as shown in Figure 5. Most private-sector hernia surgeries are concentrated in just a few counties, with Bucharest (280 cases), Constanța (185 cases), and Iași (146 cases) dominating. Strikingly, in 26 counties, not a single private hospital performed a hernia repair in 2023, raising concerns about the availability of private surgical services in large parts of the country.

**Figure 6:**
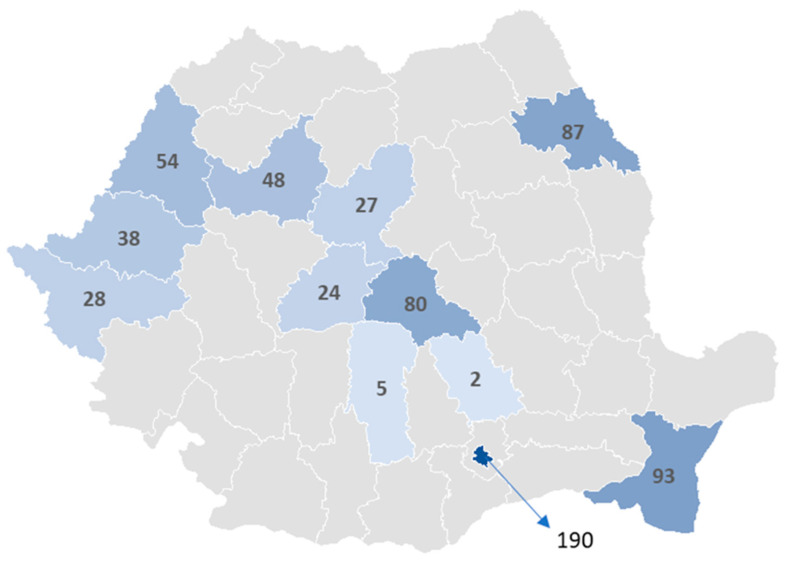
Territorial distribution of laparoscopic groin hernia repair case in private hospitals in 2023.

A similar pattern is observed for laparoscopic procedures in private hospitals, with Bucharest (190 cases), Constanța (93 cases), and Cluj (80 cases) leading. In many counties, minimally invasive procedures remain rare or nonexistent, highlighting the inequality in access to laparoscopy across private healthcare (Figure 6).

Another key finding is that private hospitals are concentrated in the same counties where the public healthcare system is already well developed. Instead of expanding services to underserved areas, they compete with public hospitals in major urban centers such as Bucharest, Cluj, Timiș, and Iași. However, Brașov and Constanța stand out as exceptions, where private surgical activity appears to fill some gaps in access to care.

### 3.3. Length of Stay

Analyzing the evolution of the mean length of hospital stay (LOS) in public and private hospitals for both open and laparoscopic surgeries, significant differences are observed between the two healthcare systems and between the surgical approaches. In public hospitals, there has been a notable reduction in LOS in recent years, particularly for open surgery. If in 2019, patients undergoing open repair stayed an average of 5.21 days in the hospital, by 2023, this duration had decreased to 4.54 days, indicating improved postoperative care and a more efficient patient flow. Similarly, for laparoscopic surgery, the hospitalization duration decreased from 3.52 days in 2019 to 2.91 days in 2023, confirming the trend of shortened hospital stays in the public sector.

**Figure 7:**
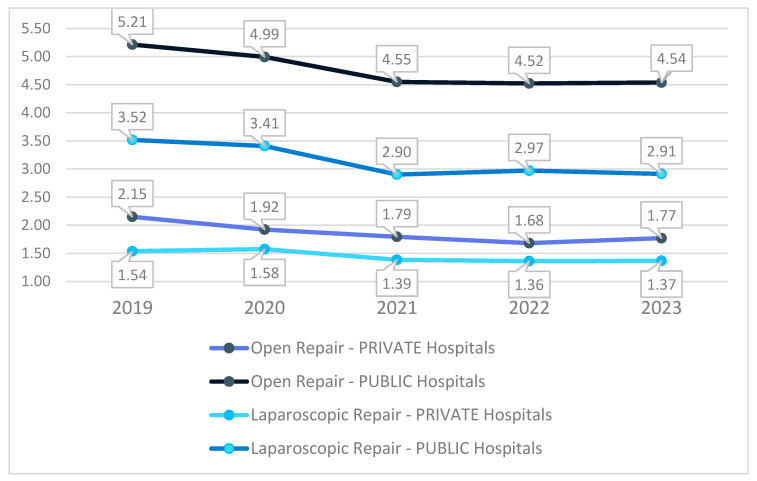
Evolution of the mean length of stay.

In private hospitals, the length of stay was consistently shorter than that in public hospitals, with fewer variations over time. For open surgery, the LOS decreased from 2.15 days in 2019 to 1.77 days in 2023, while for laparoscopic surgery, values remained relatively stable, ranging between 1.36 and 1.58 days throughout the analyzed period (Figure 7).

The main reason for the substantial differences between the two systems can be observed when analyzing the timing of the surgical intervention relative to the patient’s admission. Data from Table 3 show that in private hospitals, patients are predominantly operated on the same day as admission, with an average of only 0.16 days of preoperative hospitalization. In contrast, in public hospitals, the waiting time before surgery is significantly longer, with patients being operated on 1.30 days, on average, after admission, meaning that the surgery typically takes place on the second or even third day of hospitalization. This longer preoperative wait significantly adds to the overall hospital stay in public hospitals.

A Mann–Whitney U test comparing laparoscopic vs. open surgery confirmed statistically significant differences (*p* < 0.001) in hospitalization duration, and preoperative and postoperative days. Laparoscopic surgery was associated with significantly shorter LOS across all hospital types. Similarly, a separate Mann–Whitney U test comparing public and private hospitals showed that hospitalization duration, both preoperative and postoperative, was significantly lower in private hospitals (*p* < 0.001). Finally, a Kruskal–Wallis test assessing the interaction effect between hospital type and surgical approach revealed statistically significant differences (*p* < 0.001), reinforcing that both surgical approach and healthcare sector independently influence hospital stay.

Another key factor is postoperative hospitalization duration. In private hospitals, patients are discharged significantly more quickly, with an average postoperative stay of 1.62 days, and an even lower one for laparoscopic surgery (1.39 days). However, in public hospitals, patients remain hospitalized for an average of 3.51 days postoperatively, almost twice as long as the duration in private hospitals. When analyzed separately, the differences become even more pronounced: open surgery patients in public hospitals stay 3.73 days postoperatively vs. only 1.89 days in private hospitals, while for laparoscopic surgery, the difference is 2.19 days in public hospitals vs. 1.39 days in private hospitals. These findings suggest a greater tendency for prolonged postoperative observation in public hospitals, either due to precautionary measures or administrative and logistical constraints delaying patient discharge.

Although laparoscopic surgery is associated with shorter hospital stays in both systems, this advantage is not fully utilized in public hospitals. If in private hospitals, the mean LOS for laparoscopic repair is 1.57 days, in public hospitals, it remains significantly higher, at 3.31 days. This suggests that, although the minimally invasive technique is applied in public hospitals as well, its benefits in reducing hospital stay are not fully leveraged.

Another observed aspect is the extremely low number of patients discharged on the same day after surgery, indicating that true one-day surgery is still not widely adopted in Romania, not even in the private healthcare system. Over the five-year period analyzed, only 212 patients from private hospitals and 181 patients from public hospitals were discharged on the same day, representing an insignificant percentage relative to the total number of interventions. Among them, in private hospitals, 101 patients underwent open surgery and 111 laparoscopic surgery, while in public hospitals, 114 patients were discharged on the same day after open surgery and only 67 patients were discharged after laparoscopic surgery.

### 3.4. Hernia Recurrence

We chose to analyze only cases where the first surgical intervention took place in 2019, ensuring an adequate follow-up period and a more reliable estimation of the recurrence rate.

In 2019, a total of 18,444 patients underwent surgery for hernias. Among them, 889 required at least one additional surgical intervention, and 29 underwent three or more reinterventions. For the latter group, it is clear that we are dealing with true recurrences. However, for those who had only two hospitalizations, the situation is more difficult to interpret.

Only 107 of the 18,444 patients operated on in 2019 had a documented diagnosis of recurrent hernia at their second or third intervention, suggesting a minimum recurrence rate of just 0.58%. We refer to this as a “minimum” rate because we identified numerous errors in the coding of recurrent hernia diagnoses, meaning that if we were to rely solely on this category of patients, the recurrence rate would be significantly underestimated.

A second challenge arises from the way the DRG coding system registers these cases. The DRG system does not distinguish between right and left inguinal hernias, meaning that patients who appear with two separate surgeries may either be true cases of recurrence or patients who required separate operations for hernias on both sides of the body. This lack of specificity introduces significant uncertainty in data interpretation.

If we were to rely strictly on the number of reoperated patients, we could estimate that the maximum national recurrence rate is (889/18,444) × 100 = 4.82%. However, this value is likely overestimated due to coding limitations. In reality, the true recurrence rate is somewhere in between this two values. Interestingly, no significant statistical differences were observed between reoperated patients who initially underwent laparoscopic surgery and those who had their first intervention performed using the open approach.

### 3.5. Mortality

In private hospitals, only two deaths were recorded—one following an open surgery and one after a laparoscopic procedure—out of a total of over 5200 patients operated on. In public hospitals, the number of deaths was higher, but this must be considered in the context of a significantly larger volume of procedures. A total of 248 patients who underwent open surgery and 12 patients who had laparoscopic surgery died, out of more than 71,000 cases.

Overall, the mortality rate over the five-year study period was 0.34%, reinforcing the safety of hernia surgery, regardless of surgical approach or hospital type. Among the comorbidities analyzed, only three were significantly associated with mortality. The risk of death was notably higher in patients with atrial fibrillation (*p* < 0.001), those with chronic kidney disease (*p* < 0.001), and those aged over 70 (*p* < 0.001). The highest mortality rate was observed in patients with atrial fibrillation over 70 years old, affecting 5% of this subgroup (*p* < 0.001).

### 3.6. Aspects Related to the Reimbursement for Inguinal Hernia Cases

Analyzing the reimbursement structure provided by the National Health Insurance House (NHIH), it is evident that there is no significant differentiation between open and laparoscopic hernia repairs in terms of financial compensation. The reimbursement is based on the DRG system, which does not appear to reflect the additional costs associated with laparoscopic surgery (Table 4).

In both public and private hospitals, the average reimbursement per case is similar, at EUR 513 in public hospitals and EUR 483 in private hospitals. When considering the surgical approach, open surgery is reimbursed with EUR 503 in public hospitals and EUR 494 in private hospitals, while laparoscopic procedures receive EUR 573 in public hospitals and only EUR 474 in private hospitals. However, only private hospitals are allowed to receive additional out-of-pocket payments from patients. This minor variation does not fully account for the higher equipment and material costs required for laparoscopic surgery, such as specialized instruments and disposable devices.

Furthermore, the case complexity index (ICM) remains comparable between open and laparoscopic surgeries across healthcare settings. This suggests that the DRG system in Romania does not distinguish between the two approaches in terms of complexity, reinforcing the need for an updated reimbursement model that considers the additional benefits and costs associated with minimally invasive techniques.

## 4. Discussion

The comparative analysis of inguinal hernia repair in Romania reveals significant differences between public and private hospitals, particularly in surgical approach selection, hospitalization duration, and reimbursement structures. The most striking contrast is the higher adoption of laparoscopic repair in private hospitals (54.7%) compared to public hospitals (14.6%), a trend observed consistently throughout the study period. This disparity suggests that private hospitals, benefiting from more flexible financing and the quicker adoption of new technologies, are at the forefront of minimally invasive surgery, while public hospitals remain largely dependent on open repair due to financial constraints and infrastructure limitations [14].

The overall trend in Romania is positive, with laparoscopic repairs increasing steadily in both sectors, reaching 20.1% of total groin hernia procedures by 2023. This is especially notable considering that countries such as Spain (5.3%) and Italy (6%) —countries with more advanced healthcare infrastructure—still report lower adoption rates [8,10]. However, Romania still lags behind nations like Denmark (65%) and Germany (52–66%), where laparoscopic repair has become the standard [15,16]. This suggests that, while Romania is ahead of many Southern European countries, further efforts are needed to bridge the gap with high-performing systems.

Hospitalization duration also differs notably between public and private hospitals, with private-sector patients having shorter preoperative (0.16 days) and postoperative stays (1.62 days) compared to those in the public sector (1.30 and 3.51 days, respectively). The efficiency of private hospitals in patient turnover reflects a more streamlined surgical process, likely aided by early scheduling, dedicated laparoscopic teams, and optimized discharge protocols. Meanwhile, public hospitals experience delays that extend hospital stays, often due to administrative bottlenecks or a more cautious postoperative management approach [17].

The benefits of laparoscopic inguinal hernia repair are increasingly recognized in both clinical outcomes and patient quality of life. A recent systematic review by Huerta and Garza [18] highlights that laparo-endoscopic techniques, particularly TAPP and TEP, have significantly evolved, demonstrating reduced rates of chronic inguinal pain (~10%) and recurrence rates to comparable to those for open techniques. These minimally invasive procedures also offer advantages such as less postoperative discomfort and quicker return to daily activities.

Further supporting these findings, a large propensity-score matched analysis from the French Hernia Registry compared laparoscopic and open hernia repairs on over 15,000 patients [19]. The study found that patients undergoing laparoscopic TAPP and TEP repairs had significantly lower one-year chronic postoperative pain (CPIP) rates compared to those receiving the open Lichtenstein repair (TAPP: 10.0% vs. 15.9%; TEP: 12.4% vs. 16.1%; both *p* < 0.01). These findings align with the meta-analysis by Guidi Lyra et al. [20], which demonstrated a 51% relative risk reduction in chronic inguinodynia for patients treated laparoscopically, further underscoring the patient-centered benefits of these techniques.

National registry data from England confirms laparoscopic techniques were associated with lower 30-day readmission rates (2.5% vs. 3.1%) and slightly lower reoperation rates (0.7% vs. 0.8%) compared to open surgery [21]. The benefits were most pronounced in bilateral hernias, where laparoscopy was used in 65.5% of cases.

While laparoscopic approaches to groin hernia repair are increasingly popular for their clinical benefits, proving their cost-effectiveness remains complex. A prospective randomized trial from Sanchinarro University Hospital in Madrid compared transabdominal preperitoneal (TAPP) repair with open Lichtenstein repair (OL) in bilateral inguinal hernias. Although TAPP was initially more expensive (EUR 1683.93 vs. EUR 1192.83), it offered improved outcomes, such as reduced postoperative pain, shorter hospitalization, and fewer complications. Furthermore, TAPP showed a higher quality-adjusted life year (QALY) gain (0.8094 vs. 0.6765), with a 95–98% probability of being more cost-effective than OL at standard willingness-to-pay thresholds [22].

Supporting these findings, another Spanish study analyzing modifications of the TAPP technique for unilateral hernias found that the use of fibrin glue reduced operative time and offered favorable cost-effectiveness despite higher initial costs [23]. Similarly, a large-scale German analysis of 916 hernia repairs found only a slight cost difference between TAPP and open procedures (only EUR 17 more for TAPP), but emphasized that patient factors such as multimorbidity, advanced age, emergency operations, and postoperative complications were the real cost drivers [24]. These studies suggest that while laparoscopic techniques might involve higher initial expenses, they can be economically justified when factoring in long-term outcomes, especially when performed electively in well-optimized patients.

The Romanian reimbursement system does not differentiate between open and laparoscopic approaches, despite the latter’s higher procedural costs [25]. The National Health Insurance House (NHIH) reimburses both procedures at nearly identical rates. This financial model disincentivizes the widespread adoption of laparoscopy in the public sector, as hospitals must absorb additional equipment costs without additional funding. By contrast, Belgium and other Western European countries provide additional reimbursement for laparoscopic procedures, ensuring that hospitals are not financially penalized for offering minimally invasive surgery [26,27].

The lack of a national hernia registry is another critical limitation in Romania. Countries like Denmark, Sweden, and Germany have used registries to track long-term surgical outcomes, leading to lower recurrence rates and improved quality control [28,29,30,31]. The Danish Hernia Database, for example, helped reduce recurrence rates by 50% nationwide by standardizing surgical quality and identifying best practices [11]. Romania, by contrast, relies solely on administrative DRG data, which lack clinical granularity and cannot track complications, recurrences, or long-term patient outcomes. A national registry would provide valuable insights into surgical efficacy, complication rates, and cost-effectiveness, which could guide policy decisions and enhance patient care delivery.

One of the key findings of this study is that recurrence rates did not significantly differ between open and laparoscopic repairs, confirming the safety and durability of minimally invasive techniques [32,33,34,35,36]. However, due to the limitations of DRG coding, true recurrence rates remain difficult to assess, as the data do not distinguish between same-side recurrences and new contralateral hernias. This limitation is common in administrative datasets, where miscoding and lack of long-term follow-up can obscure accurate recurrence reporting [37].

From an international perspective, Romania has achieved a remarkable adoption of laparoscopic hernia repair despite systemic barriers, demonstrating that training programs and surgeon enthusiasm can drive change even in resource-limited settings [38,39]. However, laparoscopic surgery remains concentrated in urban centers, with major university hospitals in Bucharest, Cluj, and Timiș performing the majority of these procedures, while smaller counties have near-zero adoption rates. This highlights a geographic imbalance in access to minimally invasive surgery, likely driven by the uneven distribution of trained surgeons and resources.

Moving forward, three key policy interventions could accelerate Romania’s transition toward a more balanced surgical approach. First, reimbursement adjustments should be implemented to recognize the additional costs of laparoscopic surgery, either by increasing DRG values for minimally invasive repairs or providing dedicated funding for laparoscopic consumables. Second, a national hernia registry should be developed, modeled after successful programs in Northern Europe, to collect long-term outcome data and optimize surgical best practices [30]. Finally, targeted training programs should be expanded to ensure that laparoscopic techniques are accessible not only in major academic centers but also in regional hospitals, where the majority of Romanian patients receive care [38].

## 5. Conclusions

Romania has demonstrated substantial progress in the adoption of laparoscopic inguinal hernia repair, outperforming many Southern European nations despite financial and logistical challenges. However, the public healthcare sector still lags behind private hospitals in minimally invasive surgery adoption, largely due to infrastructure constraints, limited surgeon training, and an outdated reimbursement system that does not incentivize laparoscopy. While patient outcomes for laparoscopic and open repairs are similar in terms of recurrence rates, the advantages of minimally invasive techniques—faster recovery, reduced pain, and shorter hospital stays—support the need for broader adoption.

To align with international best practices, financial incentives should be restructured, national data collection should be improved through a hernia registry, and surgical training should be expanded beyond universitary centers. The current trend in Romania is encouraging, and with the right policy changes, the country has the potential to further improve access to high-quality, minimally invasive hernia repair, ensuring equitable access to the latest advancements in surgical care for all patients.

## Figures and Tables

**Table 1:** Evolution of surgical procedures by surgical approach and hospital type.

	2019	2020	2021	2022	2023	Total
Private Hospitals	803	881	1374	1105	1094	5257
Open Repair	369	405	667	521	418	2380
Laparoscopic	434	476	707	584	676	2877
% Laparoscopic	54.05%	54.03%	51.46%	52.85%	61.79%	54.73%
Public Hospitals	17,641	9404	11,442	15,560	17,249	71,296
Open Repair	15,480	8138	9781	13,238	14,254	60,891
Laparoscopic	2161	1266	1661	2322	2995	10,405
% Laparoscopic	12.27%	13.48%	14.51%	14.94%	17.39%	14.61%
Total	18,444	10,285	12,816	16,665	18,343	76,553
% Laparoscopic	14.07%	16.94%	18.48%	17.44%	20.01%	17.35%

**Table 2:** Public vs. private hospitals: logistic regression comparison.

Variable	Public OR (95% CI)	Public *p*-Value	Private OR (95% CI)	Private *p*-Value
Intercept	1.08 (0.93–1.25)	0.153	2.94 (2.45–3.54)	<0.001
Age	0.97 (0.96–0.98)	<0.001	0.98 (0.97–0.99)	<0.001
Male sex	1.05 (0.96–1.16)	0.256	1.20 (0.98–1.45)	0.084
Femoral hernia	0.68 (0.57–0.82)	<0.001	0.66 (0.40–1.09)	0.074
Recurrent hernia	1.21 (1.10–1.34)	<0.001	0.53 (0.42–0.67)	<0.001
Obstruction	0.64 (0.57–0.72)	<0.001	0.71 (0.57–0.89)	<0.001
Gangrene	0.84 (0.64–1.11)	0.205	1.54 (0.83–2.89)	0.172
Bilateral hernia	2.25 (1.98–2.56)	<0.001	2.18 (1.75–2.72)	<0.001
Obesity	0.95 (0.85–1.07)	0.206	0.75 (0.57–0.98)	0.037

**Table 3:** Length of hospital stay, preoperative, and postoperative days by surgical approach and hospital type.

	Mean LOS	Mean Preop Days	Mean Postop. Days
**Private Hospitals**	1.78	0.16	1.62
Open Repair	2.03	0.14	1.89
Laparoscopic	1.57	0.18	1.39
**Public Hospitals**	4.80	1.30	3.51
Open Repair	5.06	1.33	3.73
Laparoscopic	3.31	1.13	2.19
**Grand Total**	4.60	1.22	3.38

**Table 4:** Average NHIH reimbursement for surgical treatment of inguinal hernias.

Hospital/Procedure	NHIH Reimbursement (Euro)	Average Case Mix Index (ICM)
**Private**	483	1.61
Open Repair	494	1.67
Laparoscopic Repair	474	1.56
**Public**	513	1.56
Open Repair	503	1.54
Laparoscopic Repair	572	1.67
**Total**	511	1.56

## Data Availability

The original contributions presented in this study are included in the article. Further inquiries can be directed to the corresponding author(s).
